# Inflammation as A Precursor of Atherothrombosis, Diabetes and Early Vascular Aging

**DOI:** 10.3390/ijms23020963

**Published:** 2022-01-16

**Authors:** Elena Barbu, Mihaela-Roxana Popescu, Andreea-Catarina Popescu, Serban-Mihai Balanescu

**Affiliations:** Department of Cardiology, Elias Emergency University Hospital, Carol Davila University of Medicine and Pharmacy, 011461 Bucharest, Romania; elena.lechea@drd.umfcd.ro (E.B.); serban.balanescu@umfcd.ro (S.-M.B.)

**Keywords:** atherothrombosis, inflammation, obesity, diabetes, klotho, NLPR3, visfatin, vascular senescence, COVID-19, SUPERNOVA

## Abstract

Vascular disease was for a long time considered a disease of the old age, but it is becoming increasingly clear that a cumulus of factors can cause early vascular aging (EVA). Inflammation plays a key role in vascular stiffening and also in other pathologies that induce vascular damage. There is a known and confirmed connection between inflammation and atherosclerosis. However, it has taken a long time to prove the beneficial effects of anti-inflammatory drugs on cardiovascular events. Diabetes can be both a product of inflammation and a cofactor implicated in the progression of vascular disease. When diabetes and inflammation are accompanied by obesity, this ominous trifecta leads to an increased incidence of atherothrombotic events. Research into earlier stages of vascular disease, and documentation of vulnerability to premature vascular disease, might be the key to success in preventing clinical events. Modulation of inflammation, combined with strict control of classical cardiovascular risk factors, seems to be the winning recipe. Identification of population subsets with a successful vascular aging (supernormal vascular aging—SUPERNOVA) pattern could also bring forth novel therapeutic interventions.

## 1. Introduction

In addition to the well-known common risk factors for cardiovascular disease (CVD), such as hypertension, obesity, dyslipidemia and smoking, inflammation plays an important role in the pathogenesis of diabetes and associated metabolic disorders, as well as in the pathogenesis of atherosclerosis and vulnerable atheroma [1,2]. There is a permanent relationship of interdependence between systemic inflammation, diabetes and cardiovascular atherosclerotic disease, so it is becoming increasingly clear that, in addition to conventional therapy, future studies need to focus on models of inhibition on various levels in inflammation pathways [3]. Inflammation has a key role both in the progression of the disease and in its consequences and complications once installed. However, a specific anti-inflammatory treatment is not yet recommended for atherosclerotic disease and its acute complications or for diabetes. Pleiotropic therapies, such as statins, with a secondary effect on predominantly local inflammation, have been shown to be superior due to this effect [4,5,6]. High cardiovascular risk is associated with a (C reactive protein) CRP > 3 mg/L, compared with patients with a low baseline CRP [7].

The risk of CVD is significantly increased in patients with diabetes, which can be both a cause and an effect of inflammation. Moreover, CVD usually occurs a decade or two earlier in people with diabetes, and the pattern of the disease is more aggressive [8,9,10]. This observation led to the idea that aging, especially vascular aging, occurs prematurely in patients with diabetes. Aging is a complex biological process characterized by a progressive decline of multiorgan structure and function, with vascular remodeling, endothelial disfunction and loss of vascular compliance as common features [11]. Along with other multiple molecular changes, a relevant feature of aging is chronic low-grade inflammation [12]. This is now termed “Inflammaging”. Type 2 diabetes mellitus (T2DM) is characterized by multiple conditions among the elderly, probably corelated with inflammaging [13,14,15,16]. Possible pathways of inflammaging involve visceral obesity, cellular senescence, NLRP3 (Nucleotide-binding oligomerization domain, Leucine rich Repeat and Pyrin domain containing 3 ) inflammasome activation and oxidative stress [17].

What do diabetes and vascular aging have in common? Diabetes is a complex metabolic disorder. Altered glucose tolerance and hyperglycemia are the main features and are the result of an absolute or relative insulin deficiency or tissue resistance to insulin action [18]. Chronic hyperglycemia associated with diabetes can lead to organ dysfunctionalities that can involve the retina, kidneys, heart, nerves and blood vessels [19,20]. Common risk factors of both diabetes and CVD have drawn attention to the fact that, at least on some levels in the pathogenic pathways, the mechanisms are similar, interfere or are additional [3]. These common risk factors and features lead to the possibility, at least in terms of prevention and primary interventions, that these two complex affections can be regarded as a unit, linked by common inflammatory mechanisms. Metabolic syndrome is defined as an aggregate of major risk factors for CVD and diabetes, such as visceral obesity, atherogenic dyslipidemia, glycemic disorders and hypertension, secondary to endothelial dysfunction [13,14,15]. Abdominal obesity is the most frequently encountered feature. Visceral obesity often leads to T2DM by associating an increased need for insulin with resistance to its effects, leading to secondary hyperinsulinemia, and over time, to beta cell dysfunction [21]. Adding to these disorders, the secretion of adipocyte cytokines (adipokines) and chronic inflammation sustained by obesity promotes vascular endothelial dysfunction, high blood pressure and atherogenic dyslipidemia. All of these mechanisms are involved in the development of cardiovascular atherosclerotic disease [22].

Research is generally focused on the detection of deleterious factors for the identification of patients at risk and also for development of therapeutic methods. These methods are directed predominantly toward the suppression of the deleterious factors. Possible protective factors, more precisely their deficiency or methods of augmentation, are less extensively studied for these conditions. This is probably justified by the primary aim to combat the main pathogenic mechanism based on harmful molecules that create chronic changes in the metabolic balance. However, some patients with diabetes, with the same diabetes disease length, same risk factors, similar control and biological profile, develop cardiovascular complications and others do not, or develop them at a later stage. What worsens the prognosis in some patients is currently being intensively researched through the study of inflammation. Phenotyping the patient at risk should take into account not only the identification of harmful factors, but also the possible protective factors against emerging disease or in the development of complications.

This review aims to describe these protective and deleterious mechanisms at play in vascular disease. We also discuss possible countermeasures concerning not only drugs but also lifestyle changes effective in the management of inflammation. The association between inflammation and diabetes, obesity, premature vascular aging and atherothrombosis in both chronic and acute (e.g., COVID-19) settings is also a topic of interest.

## 2. Inflammation and Atherothrombosis

Inflammation is the main pathophysiologic mechanism leading to initiation, progression and thrombotic complications of atherosclerosis. In opposition to the innate natural defense mechanisms that protect us against external foes, such as those from bacteria or viruses, the immune system may react against various self-antigens. This leads to various chronic conditions, such as auto-immune and auto-inflammatory disease, responsible for morbidity, mortality and disability in modern society. However, the most worldwidespread inflammatory reaction against the inner self is atherosclerosis.

### 2.1. Inflammation as a Precursor of Atherosclerosis: A “Bird’s Eye View”

Extremely different factors have been identified in initiating and sustaining the inflammatory process of atherosclerosis (Table 1).

All these factors may be grouped in two different classes. PAMPs (Pathogen-Associated Molecular Patterns) are represented by different molecular components of germs, such as chronic endotoxinemia [23,24] or viral ribonucleic acid (RNA) [25,26]. DAMPs (Damage-Associated Molecular Patterns) are related to self-antigens, such as oxidized low density lipoprotein-(ox-LDL), non-stranded deoxyribonucleic acid (DNA) and RNA fragments or glycated proteins [27]. Some of the most fascinating DAMPs are the NETs (Neutrophil Extracellular Traps), either genetically determined, as demonstrated in clonal hematopoiesis of indeterminate potential (CHIP), or simply exposed by activated or dying neutrophils [28]. These are a mesh of destructured DNAstrands liberated form neutrophils, containing tissue factor, myeloperoxidases and other proteinases. They are a major link between innate immunity, inflammation and coagulation resulting in atherothrombosis [29].

**Figure 1:**
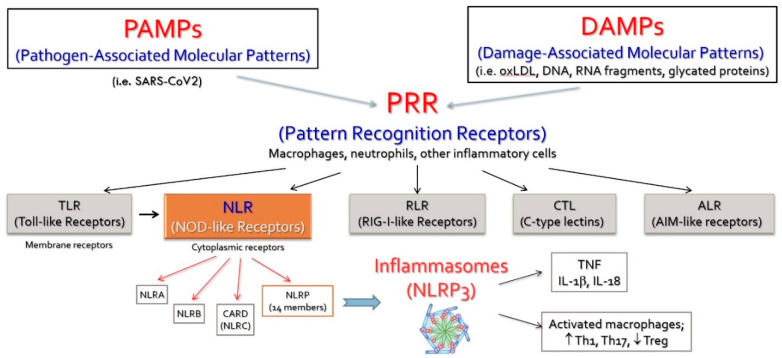
The systemic effects of NLRP3 caspase-1-mediated interleukin activation. Interleukin-6 generated by the potent stimulus of IL-1b leads to secretion of acute-phase proteins in the liver, induces endothelial dysfunction and activates smooth muscle cells in arterial media. IL—interleukin; ox-LDL—oxidated LDL molecule; CRP—C-reactive protein; PAI-1—plasminogen activator inhibitor-1. (Reproduced with permission, [31]).

Both PAMPs and DAMPs will interact with dedicated receptors widely distributed on inflammatory cells, such as macrophages, neutrophils and dendritic cells. Five classes of these receptors (PRRs—Pattern Recognition Receptors) have been described [30]. They are distributed either on the cell membrane (TLRs—Toll-like Receptors) or in the cytoplasm (NLRs—NOD-like receptors) (Figure 1).

**Figure 2:**
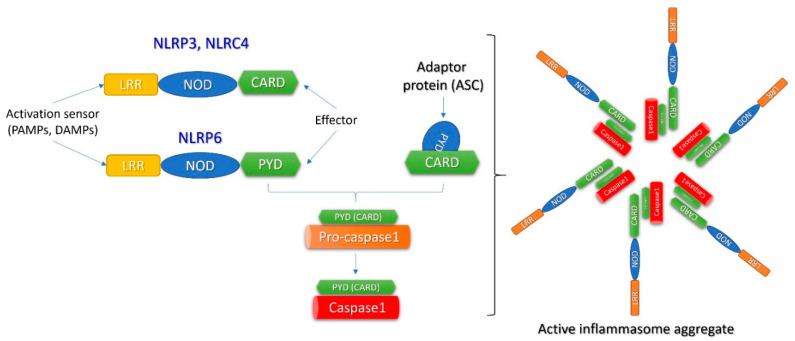
The molecular structure of the inflammasome. This is a large complex multimeric protein consisting of oligomerized NLRP. This consists of a central NOD immune receptor, a leucine-rich repeat (LRR) domain acting as an activation sensor and an effector domain, either a pyrin-containing (PYD) or a caspase activation and recruiting domain—CARD. When activated, the effector domain of NLRP interacts with an adaptor protein (ASC) via a CARD molecule and activates pro-caspase1 into mature caspase1. Pro-caspase1 also has a CARD molecule allowing interaction with the inflammasome. (Reproduced with permission, [31]).

**Figure 3:**
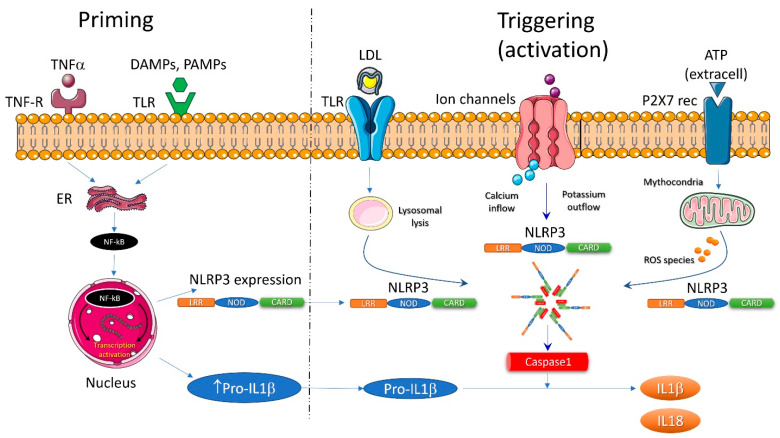
The two main steps for full inflammasome activation. Initiation of the process is priming, which leads to increased expression of NLRP3 and pro-IL-1b via nuclear factor-kappa B. This acts as a second messenger for toll-like receptor activation via DAMPs or PAMPs; an alternative pathway is NF-kB stimulation by TNF receptor and TNF α. Triggering or full activation of the inflammasome is the final oligomerization of NLRP3 with procaspase-1 cleaving properties. It can be induced by intracellular signaling due to increased ROS generation by dysfunctional mitochondria, potassium efflux or calcium influx, or lysosomal lysis after LDL crystal endocytosis. (Reproduced with permission, [31]).

The NLR family consists of fourteen different molecules that have been named as “inflammasomes” [32]. The most studied inflammasome is NLRP3. It exists in the cytoplasm as an inactive monomer (Figure 2). Its intracellular expression is increased by an nuclear factor kappa-light-chain-enhancer of activated B cells (NF-kB) -mediated mechanism after activation of TLRs by DAMPs or PAMPs and/or by Tumor necrosis factor (TNF)receptors stimulated by TNF-alpha (the “priming” process). Subsequently, NLRP3 monomers are oligomerized in the active NLRP3 inflammasome in the “triggering” process (Figure 3) [33]. As far as DAMPs and PAMPs are structurally extremely different, they could not directly interact with the NLRP3 monomers in the cytoplasm to set off their oligomerization. Different activation pathways are investigated for NLRP3 triggering: lysosomal lysis, mitochondrial generation of reactive oxygen species (ROS), calcium inflow or potassium outflow [34].

The consequences of NLRP3 activation are many and deleterious. When activated, NLRP3 inflammasome contains caspase-1 that will cleave pro-interleukin-1-beta and pro-interleukin-18 in interleukin-1-beta (IL1- β) and interleukin-18 (IL-18) [35]. These potent pro-inflammatory cytokines are responsible for inducing pyroptosis, a type of cell death resulting in supplemental inflammation [36].

Both Il-1-β and Il-18 stimulate production of interleukin 6 (IL6) from activated macrophages. Il-6 will stimulate hepatocytes into synthesizing CRP, plasminogen activator inhibitor-1 (PAI-1)and fibrinogen, activate endothelial cells to express adhesion molecules and smooth muscle cells in tunica media [37].

Activation of NLRP3 leads to proliferation of macrophages, (T helper type 1 (Th1) and T helper type 17 (Th17) lymphocytes and may amplify serum TNF-alpha concentration [38]. Plasmacytoid dendritic cells found in the lymphoid tissue surrounding the arterial wall may also be activated by the NLRP3 inflammasome and contribute to Th1 and Th17 amplification while inhibiting the anti-inflammatory regulatory T (Treg) lymphocytes [39].

Finally, activated NLRP3 is responsible for generation of more DAMPs in a vicious circle of inflammatory changes [40].

NLRP3 was not only identified in the major inflammatory cells (foam cells, dendritic cells or neutrophils) but also in the dysfunctional endothelium, in myocardial cells or in myocardial interstitial fibroblasts [41].

### 2.2. Atherosclerosis as an Inflammatory Disease

Some of the main DAMPs responsible for inflammation in atherosclerosis are serum lipoproteins. Irrespective of their serum pattern (LDL, Lp(a) or apoB, apoB100-containing lipoproteins are well-known endothelial offenders that initiate the atherosclerotic process.

The main lipoprotein of atherosclerosis, LDL cholesterol, may activate its own specific pattern recognition receptor, the CD36. The internalized LDL-CD36 complex is degraded via lysosomal endocytosis, an intracellular pathway for NLRP3 activation [42]. In reverse, activated NLRP3 inflammasome may dissociate LDL crystals in the cytoplasm of foam cells [43].

In chronic coronary syndromes, NLRP3 is correlated with the severity of coronary atherosclerosis [44]. Patients with coronary atherosclerosis show increased NLRP3 expression in blood monocytes, mainly during an acute coronary event [45]. Myocardial necrosis in acute coronary syndromes releases cellular debris, which may act as DAMPs to activate NLRP3. Myocardial injury due to coronary reperfusion also leads to mitochondrial dysfunction and ROS production, an alternate pathway of inflammasome activation in the cell [46]. As far as inflammasome activation occurs in two phases, it is presumed that in acute coronary syndromes ischemic injury is responsible for priming the NLRP3 and pro-IL-1β by locally released DAMPs. In the second phase, reperfusion triggers inflammasome activation by mitochondrial damage and ROS generation [41].

A recent large gene study enrolled 538.167 subjects and demonstrated that carriers of the intronic NLRP3 variant rs10754555 (a “gain of function” defect) had high levels of CRP and SAA (serum amyloid A) as surrogate markers for inflammation. These subjects had a higher prevalence of cardiovascular events and mortality [47].

Activated NLRP3 is an independent predictor of major adverse cardiovascular events in atherosclerosis and is independently associated with cardiovascular outcome [48]. Data from recent clinical trials suggest that NLRP3–IL1 β pathway inhibition may prove beneficial in atherosclerotic CVD [49,50].

Statins reduce NLRP3 expression in blood mononuclear cells of patients with coronary artery disease [51,52]. The effect is assigned to reduction of serum LDL levels but also to a reduction of LDL load of macrophages. LDL efflux pathways may inhibit NLRP3, neutrophil cellular traps formation and may reduce atherogenesis in experimental studies [53]. Colchicine, a potent non-specific anti-inflammatory drug, also lowers NLRP3 expression and serum levels of IL-1β and IL18 in patients with acute coronary syndromes [54]. Significant reduction of inflammation may be one of the “pleiotropic effects” responsible for clinical benefit of statins and colchicine, widely proven in multiple randomized controlled trials (RCTs). The monoclonal antibody canakinumab, an anti-IL-1β agent, demonstrated significant reduction of CRP and improved CV outcome in patients with acute coronary syndromes [55].

### 2.3. Atherosclerosis and Thrombosis: The Inflammatory Link

There is a major interaction between inflammation and thrombosis, all started from DAMPs, PRR activation and NLRP3 triggering. Inflammasomes act as amplification pathways not only for atherogenesis, but also for thrombosis [56].

Il-1β and Il-18 generated by NLRP3-caspase activation enter a self-amplification loop and also induce 1l-6 generation by the macrophages. Il-6 stimulates hepatocytes to produce CRP, fibrinogen and PAI-1, releasing them systemically and mediating the propensity for thrombosis. Il-6 directly potentiates another prothrombotic pathway mediated by JAK1/TYK2 membrane receptor leading to thrombocytosis and procoagulant activity [57].

DAMPs as ox-LDL activate platelets contributing to development of a systemic prothrombotic state [58]. LDL platelet complexes interact to activate monocytes and amplify inflammation [59].

However, among all DAMPs, the most relevant ones for “inflammatory thrombosis” are the NETs [29]. They act with the platelets and monocytes to induce thrombosis. The degenerated DNA mesh, extruded by either genetically programmed or dying neutrophils, acts as thrombosis promoter at the endothelial interface [60]. NETs may contain and activate tissue factor to initiate coagulation by the extrinsic pathway. They also directly activate Factor XII to promote contact pathway of clotting. NETs directly activate platelets through their histone content and bind to the von Willebrand factor. Last but not least, NETs contain myeloperoxidases that may cleave coagulation factors and inhibit endogenous anticoagulants [61].

Above all, inflammatory pathways may activate thrombosis in atherosclerosis but also in immune-mediated inflammatory diseases (a process called immuno-thrombosis), such as systemic lupus erythematosus, rheumatoid arthritis, psoriatic arthritis or inflammatory bowel diseases. [62].

Clinical trials with different agents targeting NLRP3 in atherothrombosis are underway [63,64]. These trials investigate whether blocking inflammatory pathways will result in superior outcomes not only in atherogenesis but also in atherothrombosis.

## 3. Inflammation and Obesity

Adipose tissue was long considered to have a single function, that of storing fat. It is now recognized as an endocrine organ that secretes various adipokines, including leptin, adiponectin, resistin and visfatin, as well as cytokines and chemokines involved in both systemic and local inflammation, such as TNFα, IL-6 and monocyte chemoattractant protein 1 (MCP-1) [65,66].m. Macrophage infiltration, the histopathological feature of inflammation of adipose tissue, is clearly correlated with body weight and adipocyte size, and the percentage of macrophages in adipose tissue is estimated to range from <10% normal weight to almost 40% in obese patients [67,68]. Hypertrophy of adipocyte cells induces necrosis leading to macrophage infiltration. A share of 90% of macrophages surround dead cells, in the shape of a crown. They phagocyte detritus and lipid droplets resulted from adipocytes’ death, forming giant cells with multiple nuclei, a symbol of chronic inflammation [69,70]. Local and systemic inflammation is promoted by macrophages’ secretion of chemokines and cytokines as MCP-1 mRNA, an important factor in vascular endothelial dysfunction and atherogenesis [71,72].

Obesity induces changes in methylation of leukocytes’ DNA, causing immune disturbances. Excessive fat tissue is also associated with mitochondrial dysfunction, increased apoptosis in adipocytes, reduced oxidation of fatty acids and metabolic low-grade inflammation [73,74].

Dietary saturated fatty acids (SFAs), for example palmitic acid (PA), increased in obese patients’ diet, are potent factors that may act as DAMPs. PA affects AMP-activated protein kinase (AMPK)–autophagy–ROS signaling axis, causing disintegration of the mitochondrial membrane and inducing ROS accumulation and NLRP3 inflammasome activation [75]. Loss of mitochondrial integrity following mitochondrial permeation transition pore (mPTP) opening is also superimposed with one of the theories of aging [76]. That leads to the deduction that NLRP3 activity is implicated in aging process.

Adiponectin, the most studied of adipokines, is a polypeptide hormone, historically known as a protective factor in diabetes and CVD. However, there are also controversies about adiponectin. Its physiological effect on inflammation and immunity is not clear enough, given that it has been shown to also have pro-inflammatory effects, stimulating the secretion of chemotactic factor and IL 6 in the fat cell [77]. It appears that different conformations of adiponectin may have different effects: those with high molecular weight (HMW) have pro-inflammatory effects, and those with low molecular weight (LMW) produce anti-inflammatory effects [78]. More recent studies show that adiponectin may have a pro-inflammatory effect in patients with auto-immune diseases. Elevated adiponectin levels have been associated with the progression of intestinal inflammatory disease and rheumatoid arthritis. In chronic inflammatory diseases with low inflammation, such as diabetes, adiponectin has low values, while in auto-immune diseases with significant inflammation, adiponectin has high values. This may indicate that adiponectin may have different effects depending on the isoform and the tissue on which it exercises its effect. The answer could come from different measurement techniques and distinction of adiponectin isoforms (LMW trimer, MMW hexamer and HMW multimer) [79].

Apart from adiponectin, another adipokine involved in modulating obesity-related inflammation is visfatin, which was found in high concentrations at the site of carotid plaques and is associated with lesion instability of coronary atherosclerotic disease [80,81,82]. Activation of the NLRP3 inflammasome has been shown to be the root cause of visfatin-induced endothelial inflammation [83].

High plasma levels of leptin determine endothelial oxidative stress and activation of the renin–angiotensin–aldosterone system “RAAS” [84,85]. Adiponectin and leptin appear to play important roles in vascular homeostasis and consequently in arteriosclerosis and hypertension [86].

There is a two-way link between NLRP3 inflammation and obesity: the metabolic imbalances related to obesity activate inflammation and, once activated, inflammation influences the prognosis. Increased expression of NLRP3, caspase-1 activity and IL-1β concentration in adipocytes and adipocyte macrophages has been demonstrated in obese patients. These findings are associated with the severity of diabetes and with every one of the abnormalities of metabolic syndrome [87]. Furthermore, elimination of NLRP3 inflammasomes in obese mice reduces IL-18 and interferon-γ (IFN- γ). IFN-γ expression in fatty tissues, increases naive lymph cell count and reduces effector T lymphocyte count [88]. Activation of these inflammasomes appears to be the explanation for chronic inflammation that characterizes insulin resistance and obesity [89]. NLRP3 inflammasome components and activation products are highly expressed in fat tissue of obese individuals, and their presence is directly correlated with the severity of the disease [90]. In the current context of SARS-COV2 infections, the connection between obesity and NLRP3 activation seems to play an important role in the outcome of COVID-19 in these patients [91]. Previous priming of the inflammasome will make these patients more vulnerable, with quicker inflammasome assembly and cleavage of Gasdermin D by caspase-1 and Gasdermin pore formation, leading to pyroptosis [91].

There is an older-age-dependent redistribution of adiposity toward the abdominal viscera that occurs unrelated to the total fat tissue mass and creates immune-metabolic disturbances, leading to the hypothesis that abdominal fat is associated with early aging features [12]. Furthermore, both IL-1 and IL-18 mediate obesity and also age-induced metabolic disturbances [92].

Obesity may cause an increase in the rate of telomere shortening, mainly due to oxidative stress and low-grade chronic inflammation, a process associated with premature aging, as it has been demonstrated that the shortening of telomere is negatively correlated with lifespan [74,93,94].

In experimental studies, diet-induced obesity was associated with premature vascular senescence [95]. This is a consequence of cellular senescence of vessel that predisposes to age-related artery diseases, such as peripheral ischemia, myocardial infarction and stroke. Furthermore, the pro-inflammatory secretory phenotype, a feature of senescence, may play a role in obesity complications, such as insulin resistance, diabetes and cardiovascular increased mortality.

## 4. Inflammation in Diabetes—What Are We Dealing With?

Researchers have identified higher levels of inflammatory markers in diabetic patients decades ago, later showing that obesity, especially visceral obesity, causes chronically elevated levels of cytokines that alter the action of insulin and contribute to the disease. Insulin resistance also results in inflammation, creating a vicious circle that facilitates disease progression and complications. The clear causal relationship between diabetes and inflammation is difficult to define, but at present, we have evidence of inflammatory cytokine involvement on several levels in the pathogenesis of diabetes. Metabolic syndrome and T2DMshare several metabolic imbalances in lipid profiles and acute phase reactants, such as creative protein (CRP), SAA, TNF alpha and IL6. Fibrinogen, tissue factor, complement component, PAI1 and tissue plasminogen activator (tPA) are also increased, but with smaller variations [96,97,98]. Obesity and T2DM are pathologies with low levels of chronic inflammation in response to the activation of innate immunity secondary to environmental, metabolic and genetic factors [65].

Accumulation of advanced glycation end products (AGEs) in tissues is a feature of aging, and their expression is increased in diabetes and renal disorders [99]. It has already been shown that endogenous AGEs, highly expressed in a diabetic environment, interfere by multiple mechanisms in vessel wall homeostasis, and their concentration is positively correlated with the extension and severity of atherosclerosis [100,101]. Their action is manifested mainly through two mechanisms: a direct one and one that is receptor dependent. The direct way is by altering the structure and function of proteins in the glycation process. AGE interaction with endothelial surface receptor for AGEs (RAGE) is a receptor-dependent mechanism. Irrespective of the action pathway, these molecules activate different cells causing excessive production of ROS and release of pro-inflammatory cytokines. Dietary AGEs exposure also triggers a strong response from the immune innate system, irrespective of the presence of diabetes or obesity. AGE exposure releases TNF-α from human macrophage-like cells in a concentration-dependent manner, induces cellular stress and promotes activation of inflammatory pathways [102,103]. Food AGEs that are not absorbed and are released in the colon interfere with local microbiota metabolism and intestinal integrity and permeability, resulting in chronic low-grade inflammation [99,104].

Hyperglycemia, high ROS concentration and the interaction of AGEs and their receptors (RAGE) within the endothelium are associated with the secretion of oxidative-mediated cytokines secretion. This consequently induces the expression of cell adhesion molecules vascular cell adhesion molecule-1 (VCAM-1), intercellular adhesion molecules (ICAM1), MCP-1 and E-selectin) in endothelial and vascular smooth muscle cells, leading to endothelium-dependent vasodilation dysfunction and promoting endothelial cell apoptosis [82,105]. In addition to glucotoxicity, lipotoxicity is also related to endothelial dysfunction.

Endoplasmic reticulum (ER) stress may be one of the pathways leading to T2DM by causing both insulin resistance and cell loss. Nutrients excess, obesity and cytokine secretion are related to ER stress [106,107].

It has been postulated that the intestinal microbiome may interact with the immune system by inducing metabolic changes that underlie the molecular origin of the low-level inflammation that characterizes obesity and diabetes [108]. Alteration of the intestinal microbiome can directly affect the immune cells within the gut and through microbial products as lipopolysaccharides (LPS), metabolites and short-chain fatty acids (SCFA), together interfering with adipogenesis and/or insulin resistance [109].

Switching the macrophage phenotype from predominantly M2 type anti-inflammatory to increased proportions of M1 type pro-inflammatory macrophages plays a crucial role in initiating and amplifying inflammation in the Langerhans islets. However, evidence shows that B and T cell recruitment precedes the infiltration of adipose tissue by macrophages [110].

In diabetes, NLRP3 activation is triggered by oxidized LDLs, free fatty acids, cholesterol crystals, ceramides and uric acid, together with reactive oxygen species, elevated serum glucose levels that act as endogenous damage-associated molecular patterns. NLPR3 activation is related to macrovascular complications, especially myocardial infarction, independent of blood glucose and cholesterol levels [111]. It is worth highlighting that T2DM is, historically, the first metabolic disease associated with NLRP3 inflammasomes [112,113].

IL1 beta production, consequent to NLRP3 activation, leads to pancreatic beta cell dysfunction and destruction [114]. In addition, IL 1 beta promotes oxidative stress in the endoplasmic reticulum and cell death, processes involved in the pathogenesis of T2DM [115]. In addition, it promotes overexpression of other inflammatory mediators, such as IL18 and IL33 signaling and enhances the inflammatory response [116].

Il 18 is activated roughly through mechanisms required for IL1 beta activation. It induces the secretion of TNFα, which is responsible for promoting IL-6 and CRP synthesis. All mentioned cytokines are associated with insulin resistance and progression ofT2DM.

NLRP3 inflammasome activity is divided into two subcategories in diabetic patients. The first subcategory includes direct recognition of diabetes-related DAMPS and activation, while the second subcategory is related to gut microbial changes and associated inflammation [113].

Regardless of the etiopathogenetic mechanisms that underlie various types of diabetes, the common pathway appears to be inflammation of Langerhans beta cells in the pancreatic islets. The concept is of an auto-inflammatory process, which results in reduction of the number of beta cells and a functional alteration [117]. The inflammasome/IL-1beta pathway, activated in the pancreatic islets, is the most common, best-studied and with the highest impact on beta cell dysfunction [118].

Experimental studies have confirmed that IL-6, along with other inflammatory cytokines, induces apoptosis in the pancreatic islets and acts as a predictor and pathogen marker for T2DM progression [119]. Immune system activation is closely linked to the incidence and progression of T2DM, and adaptive and innate immunity is involved in inflammation of adipose tissue.

There is a well-studied relationship between glucose metabolism abnormalities, diabetes and IL-1β-mediated NLRP3 activation. Further studies need to deepen the research on the effect of NLRP3 inhibition in diabetes. It is necessary to identify the cross-link and balance between beneficial and detrimental inflammasome activations for new therapeutic approaches that suppress NLPR3.

The presence of obesity and diabetes is associated with early vascular aging, even among adolescents [120]. Inflammation-derived deleterious mechanism seems to be responsible for all the features of vascular early decline (see Table 2).

## 5. Protective Factors in Diabetes and Cardiovascular Disease

### 5.1. Klotho Proteins

Klotho is a membrane-soluble protein implicated in the proper functionality of many organs. It has an anti-aging and cardioprotective role. There is important evidence that Klotho deficiency correlates with the onset and development of coronary diseadeatherosclerosis, myocardial infarction and left ventricular hypertrophy [142]. Therefore, the involvement of Klotho in signaling and regulation of adequate cellular metabolism may have an important role in cardiac and vascular protection. There are three subfamilies of the Klotho protein group, namely α-klotho, β-klotho and γ-klotho. α-klotho activates fibroblast growth factor (FGF) 23, while β-klotho activates FGF19 and FGF21. The Klotho protein group with its receptors is found on the surface of tissue cells. Their action is closely associated with that of a group of hormones—FGFs. When the subfamily is not specified, it generally refers to α-klotho. α-klotho comes in two forms: a transmembrane protein and a soluble endocrine factor detectable in blood, urine and cerebrospinal fluid. The soluble form is predominant. The transmembrane protein acts as a coreceptor for FGF 23 [143]. Its principal role is in the homeostasis of mineral metabolism. The main source is the kidney. It is also present in other organs and vessels where it exerts mechanisms of action independent of phospho-calcium metabolism and independent of renal impairment. Low levels of Klotho are related to increased mortality and morbidity associated with CVD [144,145] Animal studies have shown that mice with low levels of s-Klotho had endothelial dysfunction, atherosclerosis, accelerated arteriosclerosis and defects of angiogenesis [142,146,147]. Klotho’s absence led to premature aging syndrome, the “Klotho-deficient mice aging model”, while in an animal model with Klotho overexpression, life expectancy increased. Klotho suppressed the expression of adhesion molecules within the rat aorta and reversed the inhibition of eNOS (endothelial nitric oxide synthase phosphorylation. Low levels of Klotho are related to vascular and valvular calcifications in rats. It stimulates the production of NO (nitric oxide) with vasodilatory effects [148]. In vivo expression of the murine “Klotho gene” (mKL) in pancreatic cells attenuated β-cell apoptosis and prevented STZ (streptzotocin)-induced diabetes [149].

In humans, plasma α-klotho is elevated and insulin growth factor 1 (IGF-I) is decreased during cardiopulmonary exercise. These findings are related to reduced endothelial dysfunction [150]. Early predictors of atherosclerosis, such as intima-media thickness (IMT) in the carotid arteries, flow-mediated dilation of the brachial artery and epicardial fat thickness, were evaluated. The results showed that low serum Klotho levels were related to increased epicardial fat and IMT and lower flow-mediated arterial dilation. Thus, lower serum levels of Klotho should be considered as an early predictor of atherosclerosis [151]. Umbilical vein endothelial cells (HUVEC) were preincubated with Klotho protein and exposed to TNF α. Klotho suppressed the expression of TNF α-induced ICAM1 and VCAM1 adhesion molecules and also inhibited TNF- α induced NF-κB activation and IkB phosphorylation—mechanisms involved in inflammation and atherosclerosis, suggesting that Klotho may play a role in mediating endothelial inflammation [152]. S-klotho is involved in the regulation of oxidative stress, inflammation and fibrosis by inhibiting insulin/IGF-1 and by its action on factor-β1 metabolism transforming growth factor beta 1 (TGF-β1). Inhibition of IGF-1 by Klotho regulates oxidative stress and reduces death. The Klotho gene is expressed in pancreatic β cells. Haplodeficiency Klotho has been related to hyperglycemia, glucose intolerance, reduced insulin deposits and β -pancreatic apoptosis [149]. Endotoxemia caused depletion of cardiac Klotho and HSP70, an anti-inflammatory molecule that prevents stress-induced cellular apoptosis. Recombinant Klotho administration preserved the myocardial expression of HSP70 and improved cardiac function within the elderly. Klotho may act as a therapeutic agent in age-related CVD [153].

β-klotho is the primary receptor related to the hormone FGF21, released within the body in association with the sensation of hunger. When FGF21 binds to β-klotho protein, the hormone stimulates glucose metabolism within the body and increases insulin sensitivity. β-klotho protein encompasses a protective role in lipotoxicity and inflammation of hepatocytes, resulting in non-alcoholic liver disease in T2DM [154].

### 5.2. Adiponectin

Unlike other adipokines, adiponectin levels are low in obese patients. It has an anti-atherogenic, anti-inflammatory, cardioprotective effect and has a role in regulating insulin sensitivity. It also inhibits gluconeogenesis and accelerates glucose uptake [76]. It has been postulated that adiponectin levels are inversely correlated with IL-18 and that it inhibits IL-1β and IL-18 secretion by suppressing LPSand PA-induced NLRP3 inflammasome activation in TH-1 cells and hepatocytes [77,78].

Low baseline adiponectin levels predict arterial stiffness (AS) in hypertensive individuals [155]. Adiponectin was identified at the site of subendothelial injuries provoked by catheters, and it is believed to have a role in the repair of damaged vessels. It is also found in other conditions with disruption of the endothelial barrier, such as the atherosclerotic lesions where it may play the same reparatory role [156]. At the level of the endothelium, adiponectin also has a role in vascular homeostasis. It activates both AMPK endothelial nitric oxide synthase) and COX-2 (Ciclooxygenase-2) pathway, followed by NO production and anti-inflammatory effect and improvement in endothelial cell function [157].

**Figure 4:**
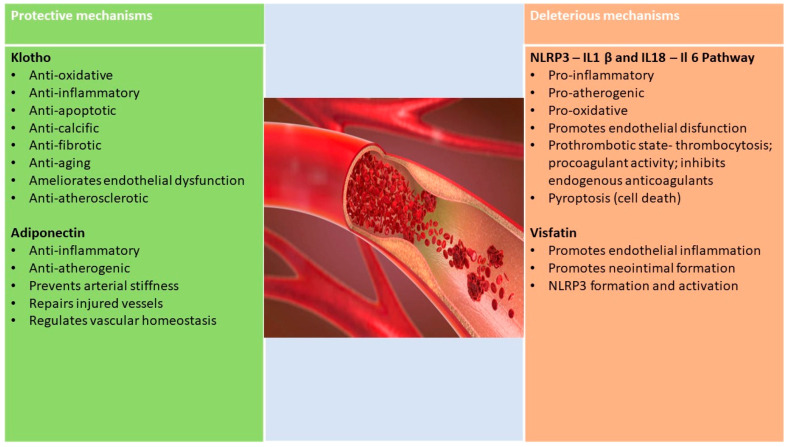
Protective vascular effects of Klotho proteins and adiponectin vs deleterious effects of NLRP and visfatin.

Thus, the long-term vascular health depends on a delicate balance between protective and damaging factors (Figure 4).

## 6. All about EVA (Early Vascular Aging)

Vascular aging is a natural process that occurs concomitantly with increasing age, through loss of elastin, increased collagen content and calcium deposition in the walls of large arteries. Early vascular aging (EVA) is, by definition, a disjunction between chronological and biological vascular age. Whether it is referred to as early vascular senescence or premature vascular aging, EVA is a distinct phenomenon associated with factors such as chronic inflammation, diabetes, hypertension or chronic kidney disease. EVA reflects an inability of the arterial wall to repair the damage produced through various mechanisms. These mechanisms induce stress on the vascular wall, either mechanical, such as hypertension, or chemical, such as acidosis in kidney disease [158] or inflammation in inflammatory bowel disease [159] and rheumatoid arthritis (RA) [160].

Early “inflammaging” causes premature medial vascular calcification leading to ASthat is intimately linked to arteriosclerosis and EVA. AS has already coined the role of intermediate cardiovascular endpoint and can be used as a predictor of CVDand mortality. AS, used as a measure of EVA, is commonly evaluated through pulse wave velocities (pWV). Carotid intima-media thickness (cIMT) is also considered to be a valuable evaluation for EVA [158].

EVA is a concept usually reserved for premature aging of the large arteries, but small arteries are also affected, showing rarefaction, increased wall thickness, bigger lumen diameter and higher wall cross-sectional area [161,162]. There is also cross-talk between the small and big arteries that creates a vicious circle of increasedAS, high central blood pressure, high peripheral artery resistance and target organ damage.

AS is hypertension independent in patients with chronic kidney disease or inflammatory disease, as shown by AS reduction with anti-TNF-α therapy, without changes in blood pressure levels [163]. AS also appears from a very young age in patients with inflammatory diseases [164]. Vulnerable individuals are at risk of early development of complications usually expected later in life. Patients suffering from RA have an increased risk of myocardial infarction, equivalent to the risk of a 10-year-older individual [158]. Identification of the types of vascular insults that the arterial wall is subjected to can lead to better prevention strategies. In this sense, screening for EVA not only in hypertensive individuals, but also in patients with inflammatory diseases, diabetes, etc., might prove beneficial.

Arterial inflammation is linked to antigens (modified lipids) that activate the immune response. T cells enter the perivascular fat and cause a pro-inflammatory environment, a process that is also amplified by chronic low-grade inflammation [165]. As NO exhibits strong anti-inflammatory effects, all conditions linked to reduced NO bioavailability through NO inactivation, dysfunction of eNOS or increased endothelin-1 are associated with AS [166].

Chronic low-grade inflammation due to chronic infections, sedentary lifestyle, physical inactivity, obesity, gut microbiota dysfunction, poor diet, socio-psychological stress, pollution or smoking is another factor that promotes long-term vascular degeneration and stiffening. Adding to chronic low-degree inflammation, high glucose levels and vessel wall protein glycation also enhance AS (as discussed above).

A recent and very elegant study demonstrates, through positron emission tomography, a significant 18F-fluorodeoxyglucose (18F-FDG) uptake in atherosclerotic plaque-free areas, suggesting that inflammation precedes plaque formation [167]. Moreover, a similar method of imaging perivascular fat inflammation was used for the coronary arteries to identify early subclinical coronary artery disease [168]. This finding confirms the relation between dysfunctional perivascular adipose tissue, inflammation and vascular disease. In physiological conditions, perivascular fat has an anti-inflammatory role, whereas age can modify its function to pro-inflammatory (e.g., endothelin-1, inducible NOS and cyclooxygenase 2) [169]. The pro-inflammatory vascular environment is associated with low-grade perivascular inflammation, creating a vicious cycle with development of AS. Another process involved in the development of AS is perivascular fibrosis. Inflammation and immune cell infiltration play a key role, mainly through Th17. Profibrotic cytokines, such as IL17, promote collagen deposition and AS [170]. It was recently shown that miR-214 regulates T cells and modulates vascular stiffening and fibrosis [170]. Recently, a metabolomic signature of EVA was described involving lysophosphatidylcholines (LPCs), compounds associated with inflammation and atherosclerosis [171]. Downregulation of four LPCs was associated with a four times higher risk of EVA [171].

Another age-related biological process of interest in this context is vascular senescence. Vascular senescence is a state of proliferative arrest associated with secretion of inflammatory chemokines, cytokines and growth factors that can affect vascular health. This phenotype, defined as the senescence-associated secretory phenotype (SASP), constitutes a link between aging, inflammation and vascular disease [131,172]. Among the secreted proteins, IL-6, IL1-α and β, IL8, IFNγ, Vascular endothelial growth factor (VEGF), ROS and TNFα are involved in endothelial dysfunction or atherosclerosis [131,173]. Vascular senescence is also produced by certain chemotherapies [174]. Vascular senescence and chemotherapy are linked through low levels of sirtuins (mainly SIRT1, but also SIRT6), proteins known to be protective molecules against inflammation and aging [175]. Moreover, chemotherapy (anthracyclines) can promote AS through vascular inflammation, inducing cytokine production ( NFκB, TGF-β, matrix metalloproteinases (MMPs), ROS, peroxynitrite and nicotinamide adenine dinucleotide phosphate oxidase (NADPH oxidase) [174].

**Figure 5:**
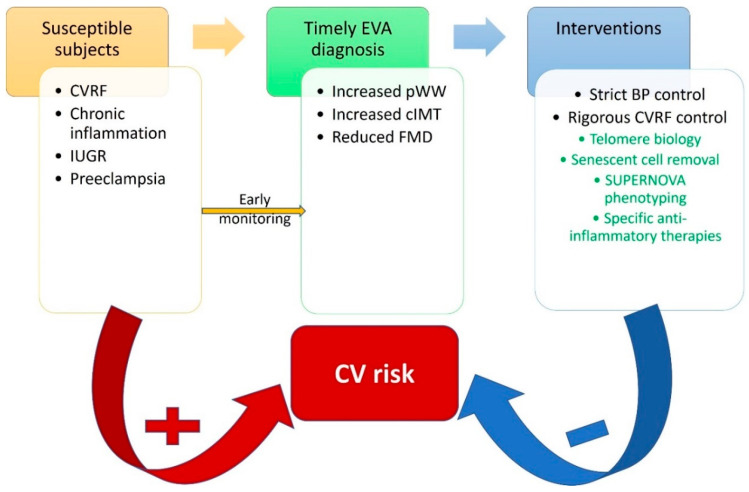
Proposed cardiovascular risk reduction strategy through identification of susceptible individuals, early diagnostic protocols and timely interventions. BP—blood pressure; cIMT—carotid intima-media thickness; CVRF—cardiovascular risk factors; FMD—flow-mediated dilation; IUGR—intrauterine growth restriction; pWW—pulse wave velocity. In green, promising future interventions.

If exposure to cardiovascular risk factors happens at a young age, vascular aging accelerates in early life [176]. For example, individuals that had intrauterine growth restriction and rapid growth after birth are a vulnerable group [136]. Thus, it is essential, given the fact that there are still many unknown or uncontrollable issues that influence the early aging process, to control exposure to factors that produce inflammation and early screening of susceptible subjects (Figure 5).

In contrast to the EVA individuals, there is a recently described population with slow vascular aging, the supernormal vascular aging group (SUPERNOVA) [177]. The SUPERNOVA phenotype has a 40% lower (6 years) vascular age and risk of cardiovascular events than individuals with the same chronological age but normal vascular aging [178,179]. This population, resilient to the normal aging process, could be a starting point in identifying factors that are key to effective arterial aging.

## 7. Man Is as Old as His Arteries—Interventions and Future Perspectives

The inevitable lifelong cumulation of factors leading to vascular dysfunction leads to exponential disease. Thus, the therapies we employ must be multifaceted as to address all of these factors. The use of risk scores, such as the Early Vascular Aging Ambulatory Score, might also help identify a vulnerable population with subclinical atherosclerosis [180].

In addition to the main mechanism of action, modern therapeutic approaches to the treatment of diabetes and atherosclerosis are also based on anti-inflammatory properties. Non-pharmacological treatments, such as lifestyle changes and pharmacological and bariatric surgical approaches to weight loss, appear to reduce systemic inflammation evaluated with CRP and IL6. Weight loss also reduces cardiovascular and all-cause mortality [181].

### 7.1. Lifestyle Modifications and Dietary Options

Choosing a proven cardioprotective diet can be useful in slowing the progression of arterial disease. The ketogenic diet has been linked to microRNA expression that modulates the antioxidant and inflammatory states in obese patients [182]. Moreover, the ketogenic diet improves the inflammatory status through weight loss, influence on the gut microbiota, insulin resistance reduction, improvement of the lipid profile [183], etc.

The inflammatory potential of 45 micro- and macro-nutrients is included in the dietary inflammatory index (DII) [184]. A pro-inflammatory score was associated with increased risk of CVD and CVD mortality [184].

Low-AGEs diet may be an effective measure for prevention and improvement of metabolic inflammation. Compared with high-AGE diet, low-AGE diet reduced TNFα, VCAM1, leptin and RAGE and increased adiponectin and sirtuin-1 levels [99,185].

The Mediterranean diet (MedDiet) has health benefits based on its anti-inflammatory, antioxidant and anti-atherosclerotic effects. Lower levels of inflammation biomarkers and reduction of cardiovascular risk factors were reported in individuals undergoing this type of nutrition [186]. MedDiet is positively correlated with telomere length and could therefore have an important role as an anti-aging lifestyle measure [187,188].

Polyphenols play a beneficial role in the prevention and development of chronic inflammation-related diseases, such as diabetes, obesity and CVD.. They modulate the immune system by lowering pro-inflammatory cytokines’ synthesis and decreasing oxidative stress and inflammatory gene expression. NLRP3 inflammasome has recently been proven to be modulated by polyphenol. Rutin, quercetin, catechin, resveratrol lychee seed polyphenol and epigallocatechin-3-gallate (EGCG) are just some of these plant-derived natural compounds that downregulate NLRP3 inflammasome activation [189,190,191,192,193,194].

The impact of physical exercise over inflammation depends on the level and type of training, and its influences are manifested through at least two mechanisms: myokines secretion and activation of laminar shear stress. It seems that the levels of anti-inflammatory cytokines are increased with regular physical activity, while in exhaustive or acute exercise, pro-inflammatory cytokines are elevated [195,196,197]. Moderate chronic physical activity is inversely corelated with decreases in expressions of TNF-α, MCP-1 and PAI-1 in adipose tissue and with 6–35% lower CRP levels [196,198]. A recently studied, Meteorin-like (METRNL) myokine enhanced during exercise seems to modulate the effect of exercise on metabolism by inhibiting IL-1β. Additionally, exercise lowers gene and protein levels of inflammasomes in fat tissue, including NLRP3 complex and its upstream inducers [199]. Physical exercise also has a beneficial cardiovascular effect through modulating the balance between protective and deleterious factors, e.g., Klotho vs IGF-1 [150]. Exercise determines laminar shear stress and downregulates the expression of endothelial angiotensin II type 1 receptor (AT1R). This reduces the formation ROS and maintains NO homeostasis. As a result, it has an anti-inflammatory and anti-atherosclerotic effect [200].

### 7.2. Statins

Justification for the use of statins in primary prevention (JUPITER) showed that rosuvastatin lowered CRP levels along with lowering LDL cholesterol, but the results for glycemic control are inconsistent. This may mean that statin use does not provide the same effect on diabetes and CVD inflammatory features [201,202]. Statins might have direct and indirect effects on adipose tissue and its secretory function, but their effects vary. For example, hydrophilic statins, such as rosuvastatin and pravastatin, have greater beneficial effects on adipokines secretion than lipophilic statins, such as atorvastatin and simvastatin [181]. The main effect is achieved by influencing the secretion of adiponectin, leptin and visfatin [203,204] and, consequently, inflammation, objectified by reduction of CRP levels [201]. Atorvastatin reduces NLRP3 activation and decreases IL1 and IL-18 levels in patients with atherosclerosis [130]. Interestingly, statins might activate NLRP3 and IFN I through decreasing intracellular cholesterol flux. Therefore, inhibition of these proatherogenic pathways (NLPR3 or IFN I) may be of assistance in patients with CVD treated with statins [205].

### 7.3. Antidiabetic Drugs

Insulin and other antidiabetic agents also have anti-inflammatory properties, evidenced by decreased levels of inflammatory markers, in addition to glycemia reduction. Lowering blood sugar alone positively influences inflammation, but intrinsic effects of currently used antidiabetic drugs have been demonstrated [10]. For example, metformin’s anti-inflammatory actions seem to be independent of blood sugar levels and more expressed on the vessels [10,206,207]. The anti-inflammatory actions of thiazolidinediones through binding and activation of PPAR gamma appear to be associated with NF-kappa-B suppression and reduced NF-kappa-B target expression [208,209,210]. A new class of antidiabetic drugs, sodium-glucose cotransporter 2 inhibitors (SGLT2 inhibitors), work by increasing renal excretion of glucose. Additional beneficial mechanisms include: decrease in production of pro-inflammatory cytokines, reduced adipose tissue-mediated inflammation, reduced oxidative stress, reduced serum uric acid, reduced glomerular hyperfiltration and albuminuria and suppression of AGE [211]. The SGLT2 inhibitor, empagliflozin, inhibits the secretion of NLRP3 and IL-1β by increasing plasma β-hydroxybutyrate and decreasing serum insulin [212,213]. Dipeptidyl peptidase-4 (DPP-4) inhibitors and Glucagon-like peptide-1 (GLP-1) receptor agonists even have intrinsic anti-inflammatory properties [135]. GLP1 agonists may intervene in inflammation mechanisms via the protein kinase A pathway in 3T3-L1 adipocytes increases adiponectin levels and decreases inflammation markers, such as IL6 [214].

### 7.4. Anti-Inflammatory Drugs

Canakinumab, an interleukin-1β inhibitor, reduced the risk of major cardiovascular events by 15% in patients with a history of myocardial infarction, randomized to 150 mg of canakinumab over standard therapy; the risk of recurrent myocardial infarction by 24%; and the risk of cardiovascular mortality by 10% [215]. For the first time, the study demonstrated that reducing inflammation decreased cardiovascular risk, regardless of LDL cholesterol levels. Although it had the same cardiovascular effects on a subset of diabetic patients, canakinumab did not reduce the incidence of diabetes.

Anakinra, an antagonist of both alpha and beta IL 1, lowers blood sugar levels, improves pancreatic beta function and reduces systemic inflammation [216]. It reduces CRP values in acute heart failure and increases exercise tolerance in heart failure with preserved LVEF [217].

Colchicine has a role in inhibiting NLPR3 inflammation and, consequently, the production of IL1 and CRP. Administration of 0.5 mg/day colchicine after myocardial infarction leads to significant decrease in cardiovascular death, myocardial infarction, stroke and emergency revascularization—compared to placebo [218,219]. Administration of 0.5 mg/day in chronic atherosclerotic coronary heart disease demonstrates a significant decrease in ischemic events [219,220].

Nebivolol improves myocardial function and lipid metabolism and decreases inflammation, possibly by inhibition of NLPR3 and improvement of mitochondrial function [221].

MCC950 is an orally administered compound that selectively inhibits NLRP3 without any effect on other inflammasomes. It is a sulfonylurea-type compound that has been shown to improve cardiovascular outcome in diabetic patients [222,223]. It has a demonstrable effect on atherosclerotic plaque reduction and improvement of vascular function by systemically reducing inflammation and oxidative stress. In diabetic patients, it reduced the activity NLRP3 and IL-1b and secondary diminished pro-inflammatory molecules derived from IL1 beta and Il 18 activation. MCC950 antiatherosclerosis effect was independent of lipidemia status [64,224]. Regarding diabetes, the administration of MCC950 improved glycemic control in allogeneic mice undergoing islet transplantation by inhibiting inflammation-responsible islet death [225].

### 7.5. Klotho Proteins

Klotho has been extensively studied in experimental models and in patients with chronic renal conditions. Klotho’s myocardial protection is determined through multiple isoform-dependent but insufficiently defined mechanisms in cardiac pathology and diabetes. Preventing the decline of Klotho, reactivating its endogenous form or administration of exogenous Klotho may serve as prevention and treatment strategies, mainly in chronic renal disease but also in diabetic patients with cardiovascular complications. Recombinant α-klotho treatment improved renal function and cardiac remodeling in chronic kidney disease. The administration alleviated renal and cardiac dysfunction induced by a phosphate-rich diet even in the absence of pre-existing chronic nephropathy [226]. Soluble Klotho may inhibit hepatic lipid accumulation in T2DM and improve insulin sensitivity [154]. Klotho treatment also reduced the expression of lectin-like oxidized LDL receptor-1 (LOX-1). LOX-1 is a major receptor for oxidized LDL in endothelial cells, a crucial step in the pathogenesis of atherosclerosis. Thus, the inhibition of the LOX-1 pathway by Klotho restricts the inflammatory response and atherogenesis [227]. Therefore, Klotho administration in the event of a cardiovascular event to limit the injury seems like a promising field for future clinical research.

## 8. Conclusions

Inflammation is intimately linked to the initiation and progression of vascular disease. Based on this certitude, phenotyping individuals at risk of developing early vascular aging might prove beneficial. Detecting molecular features of successful vascular aging can also help advance our therapeutic perspectives. Looking for therapeutic strategies to stop or slow the progression to manifest disease is the key in controlling the epidemic of cardiovascular disease. For now, joining lifestyle modifications, dietary changes and anti-inflammatory drugs with conventional therapies begins to look like a winning combination.

## Figures and Tables

**Table 1:** External or endogenous factors may act as DAMPs or PAMPs to stimulate membrane (TLRs) or cytoplasmic receptors NLRs) of monocytes, macrophages, neutrophils or dendritic cells. They may trigger inflammasome oligomerization and activate NLRP3 macromolecule.

Environmental Factors	Sympathetic Imbalance	Standard Risk Factors	Hematological Disorders	Hemodynamic Factors	Infection and Chronic Inflammation
Noise	Mental stress (acute and chronic)	LDL, Lp(a, apoB100	CHIP (Clonal Hematopoiesis of Indeterminate Potential)	Abnormal blood flow (low shear stress)	Chronic endotoxinemia (“Leaky gut sdr”)
Small particle air pollution	Insomnia and sleep deprivation	Diabetes	Efferocytosis		Gut microbiome
Diet (high fat, high sodium, low fiber)	Afferent renal nerve hyperstimulation	Smoking	Anemia, chronic hypoxia		Chronic infection (i.e., parodontosis)
		Hypertension	High blood viscosity (i.e., polycythemia vera)		
All act as DAMPs or PAMPs → Pattern Recognition Receptors (TLR, NLR)					
Inflammasome (NLRP3) activation					

LDL-low density lipoprotein, Lp(a)-Lipoprotein a, apoB100 -apolipoproteinB100.

**Table 2:** Mechanistic links between diabetes and vascular aging.

Diabetes Features Involved in Vascular Aging	Inflammation-Associated Pathways
Arterial stiffness	Chronic low-grade inflammation [121,122]; medial calcification, secondary hyperparathyroidism [123], FGF23-Klotho axis [124,125,126]; dysfunction of endothelial NO synthase (eNOS) [127]
Hemodynamic ageing, hypertension	Endothelium-dependent vasodilation dysfunction [122,127]; NLRP3-induced pyroptosis [128]; mechanical wall stress
Microvascular and endothelial dysfunction	Cytokine-induced endothelial cell apoptosis; endothelium-dependent vasodilation dysfunction [127]; accelerated atherosclerosis; local tissue renin–angiotensin–aldosterone system (RAAS) in the vascular tissue [12]; free-radical-caused abnormalities in prostacyclin (prostaglandin I_2_) secretion [129]
Chronic inflammation (general and perivascular)	NLPR3 activation, Il 1 beta, Il 18, Il 6 production; production of acute-phase proteins in the hepatocyte (fibrinogen, PAI-1, SAA, CRP); increased fibrinogen, TNF α, increased oxidative stress and adhesion molecules (ICAM1, VCAM1) [31,38,112,114,130]
Insulin resistance	Excessive production of reactive oxygen species (ROS) [10,19,90,113,131]; saturated fatty acids; alteration of the intestinal microbiome [132]; endoplasmic reticulum (ER) stress [16,112]
Defects in incretin function	Decreased NO production [133,134,135]
Hyperglycemia, AGE	Products of reaction of proteins with reactive oxygen species-advanced glycation end products (AGE). Exposure to AGE—modified protein-induced inflammatory cytokines and endothelial dysfunction oxidative-mediated cytokine secretion [82,105]
Dyslipidemia	Low-density oxidized lipoprotein (LDL)
Early-life influences	Birthweight, genetics, fetal programing [136]
Telomere length	Telomere shortening/attrition [94]
Dysbiosis of the gut microbiota	Promotion of oxidative stress-mediated arterial dysfunction [137,138]
Arterial media calcification in nephropathy	Secondary hyperparathyroidism [123], FGF23-Klotho axis [124,125,126]; hyperphosphatemia-induced endothelial cell dysfunction and apoptosis
Neuropathy and autonomous nerve dysfunction	Increased sympathetic activity; glycosylation end products; impaired circulation (enhanced vasoconstriction) [139]
High levels of uric acid	Increased vasoconstriction-cell anoxia; stimulation of renin–angiotensin–aldosterone system; perturbated dilation of the vessels due to inhibition of nitric oxide [140,141]
Oxidative stress	Increased expression of angiotensin II type-1 (AT1) receptor [12]

AGE—advanced glycation end products; AT1—angiotensin II type-1; CRP—C-reactive protein; eNOS—endothelial nitric oxide synthase; ER—endoplasmic reticulum; FGF23—fibroblast growth factor 23; ICAM1—intercellular adhesion molecule 1; Il 1 beta—interleukin 1 beta; Il 18—interleukin 18; Il 6—interleukin 6; LDL—low-density lipoprotein; NLRP3(Nucleotide-binding oligomerization domain, Leucine rich Repeat and Pyrin domain containing 3; NO—nitric oxide; PAI-1—plasminogen activator inhibitor 1; RAAS—renin–angiotensin–aldosterone system; ROS—reactive oxygen species; SAA—serum amyloid A; TNF α—tumor necrosis factor; VCAM1—vascular cell adhesion molecule 1.
